# Disease Progression Detection via Deep Sequence Learning of Successive Radiographic Scans

**DOI:** 10.3390/ijerph19010480

**Published:** 2022-01-02

**Authors:** Jamil Ahmad, Abdul Khader Jilani Saudagar, Khalid Mahmood Malik, Waseem Ahmad, Muhammad Badruddin Khan, Mozaherul Hoque Abul Hasanat, Abdullah AlTameem, Mohammed AlKhathami, Muhammad Sajjad

**Affiliations:** 1 Department of Computer Science, Islamia College Peshawar, Chartered University, Peshawar 25000, Pakistan; jamil.ahmad@icp.edu.pk (J.A.); muhammad.sajjad@icp.edu.pk (M.S.); 2 Information Systems Department, Imam Mohammad Ibn Saud Islamic University (IMSIU), Riyadh 11432, Saudi Arabia; mbkhan@imamu.edu.sa (M.B.K.); mhhasanat@imamu.edu.sa (M.H.A.H.); altameem@imamu.edu.sa (A.A.); maalkhathami@imamu.edu.sa (M.A.); 3 Department of Computer Science and Engineering, Oakland University, Rochester, MI 48309, USA; mahmood@oakland.edu; 4 Lady Reading Hospital-Medical Teaching Institute, Peshawar 25000, Pakistan; waseem.ahmad.kmcite@gmail.com

**Keywords:** disease progression, deterioration prediction, feature extraction, sequence learning

## Abstract

The highly rapid spread of the current pandemic has quickly overwhelmed hospitals all over the world and motivated extensive research to address a wide range of emerging problems. The unforeseen influx of COVID-19 patients to hospitals has made it inevitable to deploy a rapid and accurate triage system, monitor progression, and predict patients at higher risk of deterioration in order to make informed decisions regarding hospital resource management. Disease detection in radiographic scans, severity estimation, and progression and prognosis prediction have been extensively studied with the help of end-to-end methods based on deep learning. The majority of recent works have utilized a single scan to determine severity or predict progression of the disease. In this paper, we present a method based on deep sequence learning to predict improvement or deterioration in successive chest X-ray scans and build a mathematical model to determine individual patient disease progression profile using successive scans. A deep convolutional neural network pretrained on a diverse lung disease dataset was used as a feature extractor to generate the sequences. We devised three strategies for sequence modeling in order to obtain both fine-grained and coarse-grained features and construct sequences of different lengths. We also devised a strategy to quantify positive or negative change in successive scans, which was then combined with age-related risk factors to construct disease progression profile for COVID-19 patients. The age-related risk factors allowed us to model rapid deterioration and slower recovery in older patients. Experiments conducted on two large datasets showed that the proposed method could accurately predict disease progression. With the best feature extractor, the proposed method was able to achieve AUC of 0.98 with the features obtained from radiographs. Furthermore, the proposed patient profiling method accurately estimated the health profile of patients.

## 1. Introduction

In late 2019, SARS-CoV-2 infections (later recognized as COVID-19) started appearing in patients across China and quickly spread to the entire world, exhibiting highly contagious characteristics [1]. Within months, the virus started a global pandemic, severely overwhelming the healthcare infrastructure, even in the developed countries [2]. During such emergency situations with unprecedented influx of patients, hospitals become severely resource constrained. In the case of novel infectious diseases such as COVID-19, initial patient assessment is usually carried out utilizing radiographic imaging such as X-rays and CT scans [3]. This initial assessment is highly crucial in determining the severity of disease in order to establish an effective triage system for patients. Radiographic scans such as chest X-rays (CXR) are a rapid way of examining patients for symptoms of lung-related problems. They are primarily used for disease diagnosis but have also proven to be an effective modality for detecting disease progression [4]. Radiologists often use successive CXRs to monitor the progression of infection in patients. Careful examination of CXRs reveal where and how much improvement or deterioration has taken place, which is then used to determine a future course of treatment. Although it may require a quick glance by an expert radiologist, in emergency situations, automated assessment of disease progression from radiographic scans can save precious time and can therefore be highly desirable.

Medical imaging is widely recognized as a vital resource for assisting radiologists to diagnose diseases and monitor their progression. Chest X-rays, due to their fast imaging speed, lower radiation, and relatively lower costs, are the most commonly used modality for the examination and diagnosis of pulmonary diseases [5]. Although other modalities such as computed tomography (CT) or magnetic resonance imaging (MRI) reveal high-resolution, detailed anatomy in 3D mode, CXRs are widely available and inexpensive and are therefore extensively used by radiologists [6]. Considering the widespread use of CXRs in COVID-19 and other infectious pulmonary diseases, researchers have widely investigated the use of image processing, machine learning, and techniques based on deep learning to interpret them. Although sufficient success has been achieved in detection of infectious diseases from CXRs, they are still regarded as the most challenging plain film to interpret correctly [7].

Advances in artificial intelligence techniques, particularly deep learning, have led to several breakthroughs in many challenging medical image analysis and interpretation tasks, including detection, grading, delineation, and even understanding of pathological disorders in radiographic scans [8]. The availability of huge volumes of imaging datasets and the superior performance of learning algorithms have enabled methods based on deep learning to surpass human experts in disease identification [9]. Regarding CXRs, deep convolutional neural networks (CNNs) have been able to achieve highly desirable performance in the detection and diagnosis of thoracic diseases as well as successfully differentiate between bacterial and viral pneumonia [10]. In the case of COVID-19 infection, early-stage CXRs reveal interstitial infiltration in peripheral lung and subpleural regions, which gradually develops into ground glass opacities, and consolidations with varying degree of densities [11]. The health of patients with COVID-19 infection can rapidly worsen from a mild to moderate or severe state in a matter of days. In such a case, it becomes necessary to monitor the progression of patients, thereby making it necessary to obtain CXRs on a regular basis. Radiologists are therefore required to investigate the progression of infection in patients by simultaneously reading the current and previous CXRs. It is a cumbersome and sometimes challenging task to spot subtle changes in both scans. Hence, an AI-assisted method to predict disease progression from successive scans becomes highly beneficial.

Radiologists compare successive radiographic scans to determine disease progression (i.e., change in disease status over time) in a particular patient. Most current prediction systems work with a single scan to determine severity of a disease and utilize that scoring to infer its prognosis. For instance, Signoroni et al. [12] developed a multipurpose network to detect COVID-19 pneumonia, segment and align lung regions, and output severity scores by dividing the lungs into six regions. A regression head was trained on a large dataset with severity scores provided by expert radiologists for the purpose of estimating disease severity. The model achieved a mean absolute error (MAE) of 1.8. In a similar study, Cohen et al. [13] pretrained a DenseNet [14] model on 18 common radiological findings from several publicly available datasets. A linear regression model was then trained on severity scores for pneumonia extent and opacity scores provided by three expert radiologists. Amer et al. [15] trained a deep learning model to simultaneously train a detection and localization model for pneumonia in CXRs. The localization maps were then used to estimate a pneumonia ratio indicating severity of infection. Although these works effectively determined disease severity, their use of a single image did not allow them to compute the relative difference in successive scans. Sun et al. [16] used time-aware LSTM to predict disease progression using demographics and laboratory tests. The outcome was computed in terms of survival and mortality. The LSTM network received these readings about patients at different times and attempted to predict survival or mortality. They exhibited that their model was capable of predicting mortality with a high degree of accuracy. Shamout et al. [17] showed that considering CXRs along with clinical tests and patient demographics was paramount in predicting the risk of deterioration in the near future. They were able to estimate a deterioration risk curve for patients that indicated the occurrence of adverse events in the future. Pu et al. [18] utilized CT scans to determine COVID-19 disease severity and progression through segmentation and registration of the lung boundary. They identified the regions with pneumonitis and assessed progression using the segmented regions. They also generated heatmaps to indicate the affected regions, which were rated as “acceptable” by the radiologists. Feng et al. [19] also studied chest CT scans along with clinical characteristics to predict disease progression. They scored each of the five lung lobes on the basis of their involvement in the infection, and the scores were then summed to obtain an overall severity score. A similar study was conducted by Zhang et al. [20] to exhibit the capability of AI in determining disease progression from CT scans. Sriram et al. [21] developed a deterioration prediction model in terms of adverse events occurring within 96 h and mortality within the same duration from single or multiple scans. They showed that utilizing the transformer-based architecture yielded the best performance when used with multiple scans. Although the use of single image to predict severity and disease progression is dominant in the existing literature, we believe that a better estimation of disease progression could be made if multiple scans are utilized simultaneously. In this regard, we propose to utilize multiple successive scans (at least two) simultaneously to predict disease progression by comparison through deep learning methods. We model disease progression as a sequence learning problem utilizing CXR-specific features from successive scans. This strategy can be used in conjunction with severity detection models, which can only predict severity scores but cannot differentiate between two CXR with similar severity scores. There can be subtle changes indicating improvement or deterioration within a particular severity class, which the existing methods cannot detect. Automated patient monitoring systems can make good use of disease progression systems that utilize successive scans to detect positive or negative changes.

The primary objectives of this study are as follows:

1. Development of a novel sequence learning strategy for successive radiographs that can detect subtle changes in CXRs and determine improvement or deterioration.
2. Propose a patient profiling method to monitor disease progression in individual patients. The method incorporates positive or negative change in radiographs compared to a reference scan, duration between scans, and patient age to model deterioration as a function of time and age.

## 2. Materials and Methods

### 2.1. Datasets

In this study, we used a large dataset from the Valencian Region Medical Image Bank (BIMCV COVID-19) [22], which contains 2465 COVID-19 CXRs from 1311 patients. From this data, we extracted 1735 CXRs of 582 patients who had multiple scans from multiple sessions along with the associated radiologist reports. The majority of patients had 2, 3, or 4 scans, whereas some patients even had 12 or 15 scans from subsequent sessions (frequency distribution of the dataset is provided as Figure S1 in the Supplementary Material). This subset was then annotated with the help of a radiologist in terms of improvement and deterioration in the associated reports and visual examination of the scans (visual changes that appear in CXR in response to COVID-19 infection is provided in Figure S2 in the Supplementary Material). There was insufficient number of samples with no change as reported by the radiologist. Therefore, the no-change assessment was made on the basis of confidence scores of the trained model such that smaller difference in both probabilities was interpreted as negligible change or no change. The dataset was split into two sets of training and validation, with proportions of 70% and 30%, respectively.

**Figure 1:**
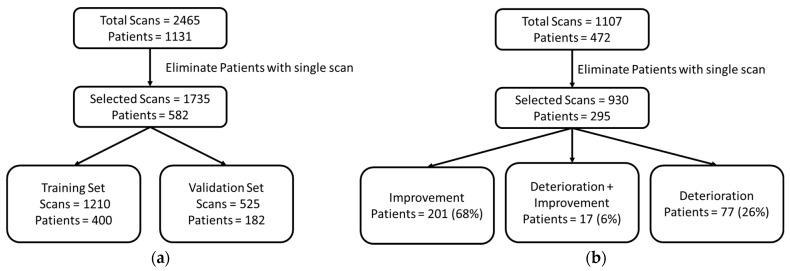
Flowchart of the study population. (**a**) BIMCV dataset used for training and validation of the models, (**b**) COVID chest X-ray dataset for testing the disease progression module.

The second dataset was the COVID chest X-ray dataset [23], which was used to evaluate the performance of the proposed disease progression detection method. From a total of 472 patients, 177 were excluded because they had only one CXR. Therefore, 930 scans from 295 individual patients were used. Each set of scans was annotated with various attributes, including scan acquisition date, age, gender, survival, need for supplemental oxygen, intubation, admitted to ICU, and others. This dataset was used to test cross dataset performance of the proposed method to determine disease progression for individual patients. It was also used to assess model robustness and effectiveness of the proposed method. Figure 1 shows the population statistics for both the datasets used in this study.

### 2.2. Proposed Progression Detection Framework

This method relies on a deep convolutional neural network to extract features from individual radiographic scans. This network should be capable of identifying a wide variety of abnormalities in CXRs. For this purpose, we trained a deep CNN to classify CXRs into mild, moderate, and severe infections of COVID-19. Features extracted by this network for a pair of scans were then fed into a long short-term memory (LSTM) network for sequence learning. This sequence was then classified into two classes of improvement and deterioration. The length of the sequence depended on the granularity of the annotations. For image level annotations, the sequence consisted of feature vectors from two images. Similarly, for lung and zone-level annotations, the sequence length was increased to 4 and 12, respectively (Zone based segmentation of CXR is provided as Figure S3 in Supplementary Material section).

The features were extracted from convolutional layers, where each neuron was sensitive to a particular visual characteristic in the image. Corresponding features could be compared to effectively learn about the changes in image characteristics. The LSTM network then determined progression of the COVID-19 infection via sequence analysis.

**Figure 2:**
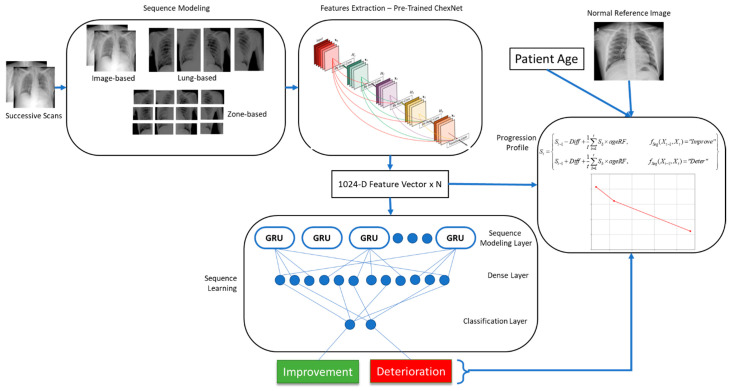
Proposed disease progression detection framework.

Disease progression detection in this work was modeled as a multiclass classification problem via sequence learning as follows:

(1) $$y=f_{Seq}(X_{t-1},X_{t})$$

where the input is a pair of successive CXRs in the frontal view [*X_t_*_-1_, *X_t_*] and the output is a label *y* ∈ {I, D indicating whether any improvement (I) or deterioration (D) is noticed in the pair of scans, and *f_Seq_* is the sequence learning function. In order to achieve this, we utilized a pretrained model ChexNet [8] to extract CXR-specific features from both images. Further details regarding each component in the proposed framework, shown in Figure 2, are provided in the subsequent sections.

### 2.3. Feature Extraction

Each scan in the pair of successive scans was separately fed to the ChexNet model for feature extraction. This network was pretrained on a large dataset containing images representing 14 different lung pathologies. The network was able to detect lung diseases at the level of an expert radiologist. For using this network as a feature extractor, the last classification layer of the network was dropped, and activations from the second last layer (7 × 7 × 1024) were obtained as feature maps. Each of these maps were generated by one of the neurons in the CNN that had become sensitive to a particular characteristic in the CXR image. The presence of that particular pattern was indicated by the position of the high activations in the map. The number of high activations in a map showed the size of that pattern in the scan. We believe that accumulating activations from each map reveals the strength of specific patterns in a particular CXR. Therefore, we used global average pooling (7 × 7) to obtain a 1024-dimensional feature vector for each image. Each value in this vector indicated the presence, absence, or strength of individual neuronal activations in the image. The output obtained from the feature extraction module is as follows:

(2) $$F_{t}=f_{ChexNet}(X_{t})$$

where *f_ChexNet_* is the feature extractor function (the ChexNet model in this case), *X_t_* is the input CXR image, and *F_t_* is the resulting feature vector. The extracted features from both scans were then constructed into a sequence, which was then utilized by the sequence modeling module for learning. The sequence was obtained as follows:

(3) $$F_{Seq}=concat(F_{t-1},F_{t})$$

In addition to extracting features from the entire image, more fine-grained features were also extracted from each scan. Firstly, each lung was segmented from both scans, and features were extracted from them individually. For both the left and right lung in each image, a separate 1024-dimensional feature vector was obtained. The sequence was then constructed by combining the extracted feature vectors as given in (4), resulting in a sequence length of 4.

(4) $$F_{Seq}=concat(F_{t-1}^{L},F_{t}^{L},F_{t-1}^{R},F_{t}^{R})$$

where $F_{t-1}^{L},F_{t}^{L},F_{t-1}^{R},F_{t}^{R}$ are the features extracted from left lung in the previous scan, left lung in the current scan, right lung in the previous scan, and right lung in the current scan. Here, the features extracted from individual lungs were considered as finer-grained and more expressive compared to the features extracted from the entire image.

Going one step further, we also subdivided each lung into three zones of upper, middle, and lower and extracted features from each zone using the ChexNet model. These features were the most fine-grained features and regarded as the most expressive ones. The sequence length in this case became 12. The final sequence in this case was constructed as follows:

(5) $$F_{Seq}=concat(F_{t-1}^{UL},F_{t}^{UL},F_{t-1}^{ML},F_{t}^{ML},F_{t-1}^{LL},F_{t}^{LL},F_{t-1}^{UR},F_{t}^{UR},F_{t-1}^{MR},F_{t}^{MR},F_{t-1}^{LR},F_{t}^{LR})$$

### 2.4. Sequence Modeling

Convolutional feature output by neurons in deeper layers of the ChexNet model were sensitive to specific properties in CXRs corresponding to the 14 abnormalities the network was trained to recognize. The labeled sequences we generated from the dataset were strictly unidirectional. If the order of the sequence were to be reversed for improvement or deterioration, their labels would also get reversed, giving rise to a new sample. Utilizing this strategy, we expanded our dataset by reversing pairs of images labeled “improvement” to obtain a sample labeled “deterioration” and vice versa. These sequences were then used to train the deep sequence learning model.

### 2.5. Deep Sequence Learning

Sequence learning is a common machine learning task, where an algorithm attempts to learn from time series data such as text sentences, speech audio, videos, and sensor readings to solve classification and regression problems. In this work, we employed deep sequence learning technique to determine improvement or deterioration in successive radiographic scans obtained over time. For this purpose, gated recurrent units were used to perform sequence learning.

The inbuilt memory mechanism implemented via gates in GRUs allow it to effectively model both short and long sequences without running into vanishing gradient problems. These characteristics make it a suitable candidate for sequence learning of subtle change detection in successive CXRs.

The sequence learning model was constructed using the ChexNet model, followed by a GRU layer, a dense layer, and finally a classification layer. The input layer took N images/patched from two successive scans depending on the granularity of feature extraction, which were input to the feature extraction network (ChexNet). This network output a 1024-dimensional vector for each image. The sequence constructed from N images of the sequence was then forwarded to the sequence learning layer consisting of 1024 GRUs. This was followed by a dense layer of 512 neurons, and finally the classification layer output probabilities for the two classes (Complete network architecture is provided in the Supplementary Material as Table S1).

### 2.6. Progression and Severity Detection

The proposed progression detection method can be used to estimate disease progression in CXRs when they are compared with known CXRs. The output of the detection model predicting either improvement or deterioration if quantified can be far more useful in determining progression. In an attempt to quantify the change in successive scans, we devised a progression estimation function (PEF) where features from the current CXR was compared with those of the previous CXR and the normal reference CXR. The inverse cosine similarity using deep features extracted from the ChexNet model was then used as a quantification mechanism for positive or negative change in both scans. An additional reference distance was also computed by taking the difference of the two CXRs from a reference normal scan. Furthermore, age has also been noted as a potential risk factor associated with COVID-19 patients. In this regard, we computed age risk factor for these patients by analyzing historical data. A risk score was computed using (6) for each age group (as depicted in Figure S4 in the Supplementary Material).

(6) $$ageRF_{a}=\frac{1}{\sum_{a=1}^{A}n_{c}^{a}}(n_{d}^{a})$$

where *ageRF_a_* is the risk factor associated with the age group *a*, $n_{d}^{a}$ is the number of COVID-19 cases in the age group *a*, and $n_{d}^{a}$ is the number of COVID-19 deaths in the group. The risk factor was simply the ratio of deaths in a particular age group to the total number of COVID-19 deaths reported for a period of time. The risk factor scores for this study were calculated from CDC data for the US [24].

This risk factor was then used as a penalty term in the PEF to determine overall progression by modeling quicker deterioration and slower recovery rates for older patients compared to younger patients. The algorithm for determining patient progression profile is provided below as Algorithm 1.
**Algorithm 1:** Progression Estimation FunctionInput: *F_ref_ =* The reference normal CXR *F_prev_* = The previous CXR *F_curr_, =* The current CXR *d =* The duration in days between the two successive CXRs *ageRF:* Age risk factor computed from historical data using Equation (10) PEF (*F_ref_, F_prev_, F_curr_, d, ageRF*) Extract features from all three input scans to construct feature vectors *F_ref_, F_prev_,* and *F_curr_* using               
$F_{X}=f_{ChexNet}(X)$ Compute inverse cosine similarity between the feature vectors as $D_{CP}=1-\mathrm{cosine}\text{\_}\mathrm{sim}(F_{curr},F_{prev})$ $D_{CR}=1-\mathrm{cosine}\text{\_}\mathrm{sim}(F_{curr},F_{ref})$ $D_{PR}=1-\mathrm{cosine}\text{\_}\mathrm{sim}(F_{prev},F_{ref})$ Compute the relative difference between successive CXRs using the weighted summation
$Diff=\frac{w_{1}.\left|D_{CR}-D_{PR}\right|+w_{2}.D_{CP}}{d}$ The *Diff* is then added or subtracted to the previous profile score based on the prediction of the sequence model, or the score remain unchanged if no change is reported.
$S_{t}=\left\{\begin{array}{l} S_{t-1}-Diff+\frac{1}{t}\sum_{b=1}^{t}S_{b}\times ageRF,\;\;\;\;\;\;\;\;\;\;\;\;\;\;\;\;\;\;\;\;\;\;\;\;\;\;\;\;\;\;\;\;\;\;f_{Seq}(X_{t-1},X_{t})=“Improve”\\ S_{t-1}+Diff+\frac{1}{t}\sum_{b=1}^{t}S_{b}\times ageRF,\;\;\;\;\;\;\;\;\;\;\;\;\;\;\;\;\;\;\;\;\;\;\;\;\;\;\;\;\;\;\;\;\;f_{Seq}(X_{t-1},X_{t})=“Deter”\\ S_{t-1}\;,\;\;\;\;\;\;\;\;\;\;\;\;\;\;\;\;\;\;\;\;\;\;\;\;\;\;\;\;\;\;\;\;\;\;\;\;\;\;\;\;\;\;\;\;\;\;\;\;\;\;\;\;\;\;\;\;\;\;\;\;\;\;\;\;\;\;\;\;f_{Seq}(X_{t-1},X_{t})=“No\;Change”\; \end{array}\right\}$ Return *S_t_*

Different sets of experiments were designed to determine optimality of the proposed framework and evaluate its performance with the optimal set of parameters. For this purpose, we experimented with a variety of feature extraction strategies from CXRs. Some of the most capable CNN architectures were evaluated as feature extractors for our sequence learning problem. Finally, the architecture of sequence learning model consisting of GRUs was optimized through experimentation.

### 2.7. Experimental Setup

The proposed framework was implemented in Google TensorFlow 2.4 (Google, Mountain View, CA, USA) with CUDA support. All the experiments were performed on a PC equipped with a 10th Gen Intel Core i7 CPU (Intel Corporation, San Jose, CA, USA) with 16 GB RAM and an Nvidia RTX 3060 GPU (Nvidia, Santa Clara, CA, USA) with 12 GB memory, running Microsoft Windows 10 (Microsoft, Redmond, WA, USA).

## 3. Experimental Results

The proposed framework was experimentally evaluated on the BIMCV COVID-19 dataset and the COVID chest X-ray dataset to exhibit its effectiveness for disease progression detection given successive CXRs of patients. The experimental setup, experiment design, and disease progression results are provided and discussed in the subsequent sections.

### 3.1. Disease Progression Results

In this experiment, we studied a wide variety of state-of-the-art CNN models for their feature extraction capabilities in radiographic scans. A number of recent deep learning models were chosen, including RresNet-50 [25], InceptionV3 [26], InceptionResNetV2 [27], ChexNet, and EfficientNet [28]. All the models except ChexNet were fine-tuned using a large dataset of COVID-19 and non-COVID-19 scans [29]. These models were then used as feature extractors to represent radiographic image sequences. To assess their performance and compare them to features from the pretrained ChexNet model, we tested these models in our image-based sequence learning experiment. The results presented in Table 1 show that the ChexNet model pretrained on 14 different pathologies in CXRs outperformed the fine-tuned models, proving that ChexNet is a superior feature extractor. Although the other models performed reasonably well, their overall performance was not on par with the ChexNet model. The performance of these models can be further improved if they are trained on COVID-19-specific abnormalities, which is an endeavor for our future research.

**Figure 3:**
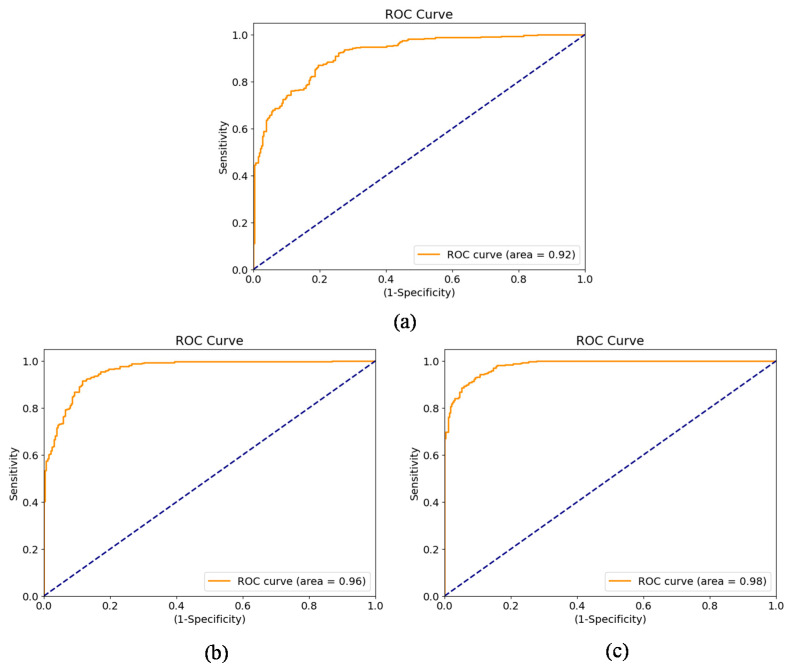
Disease progression performance with (**a**) image-based sequence learning, (**b**) lung-based sequence learning, and (**c**) zone-based sequence learning. [The dotted blue line (added for reference) is the ROC Curve of the random guess, representing no predictive capability].

In the second experiment, we tested the performance of the ChexNet feature extractor with different feature extraction strategies as reported in Table 2. For image-based scheme where the sequence length was 2, an AUC score of 0.92 was obtained. Significant improvement was observed when lung-based and zone-based schemes were used to construct longer sequences. The finer-grained features were found to be more expressive, so significant improvement was noticed over the coarse-grained features in the image-based scheme. We observed AUC of 0.96 for lung-based and 0.98 for zone-based feature extraction schemes. The receiver operating characteristics (ROC) curves for all three schemes is shown in Figure 3.

In this experiment, we evaluated a variety of sequence learning architectures and units, including LSTM and GRU. Their width and depth were also evaluated to determine the optimal architecture for the disease progression detection problem. The architectures along with their performance are shown in Table 3. The first configuration consisted of 1024 GRUs followed by dense layer of 512 neurons and a classification layer based on two neurons. Its performance was recorded to be the optimal one with an AUC of 0.98 on the test set. In the second configuration, the number of GRUs was reduced by 50%, with a 7% decrease in performance. We also experimented with two layers of GRUs with a dense layer based on 128 neurons, but the model overfit. Finally, we tested reducing the neurons in the dense layer by 50% to witness 4% decrease in performance. Although the architecture evaluation was not exhaustive, it would be interesting to see automatic configuration construction using Auto-ML methods.

### 3.2. Patient Progression Profile

**Figure 4:**
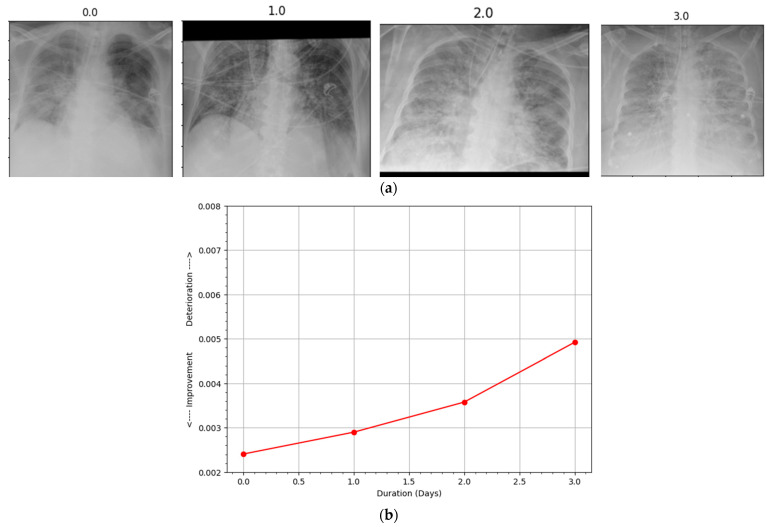
(**a**) Subsequent radiographs (178) showing deterioration, which is reflected in the (**b**) disease progression profile (represented by the red line). The patient did not survive the infection.

**Figure 5:**
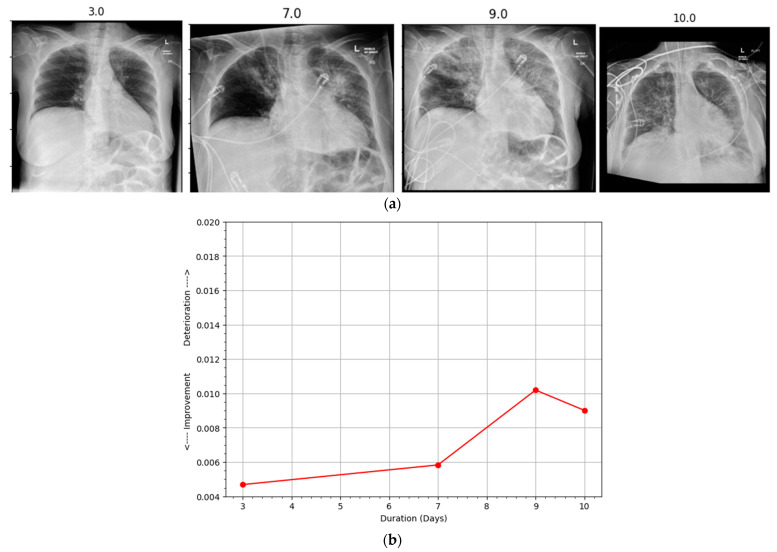
(**a**) Subsequent radiographs belonging to a patient (173) who exhibited deterioration till day 9 but showed improvement on day 10, which is reflected in (**b**) the disease progression profile (red line). The patient was intubated but survived the infection.

**Figure 6:**
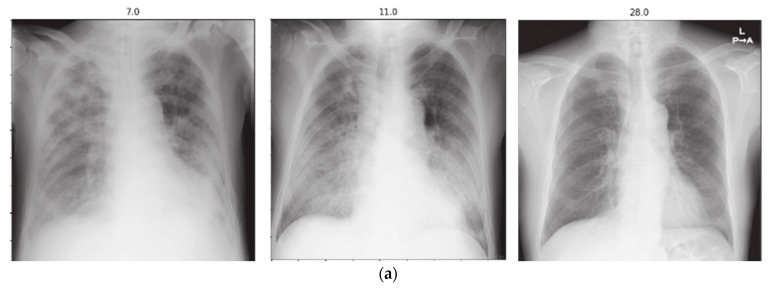

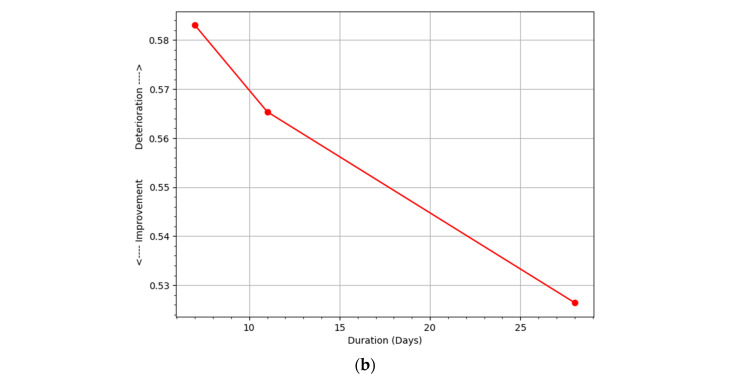
(**a**) Subsequent radiographs belonging to a patient (303) who exhibited improvement as reflected in (**b**) the disease progression profile. The patient was intubated but survived the infection.

**Figure 7:**
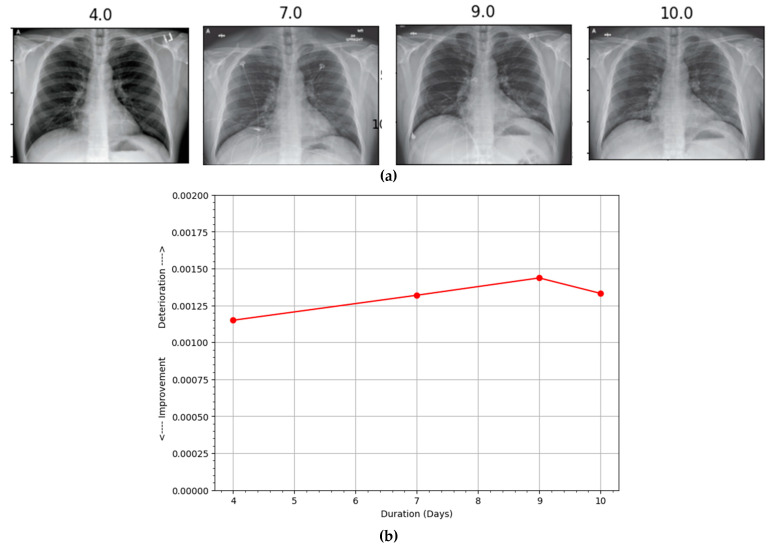
(**a**) Subsequent radiographs belonging to a patient (13) who exhibited deterioration in the scans obtained at days 4, 6, and 9 but slight improvement on day 10 as reflected in (**b**) the disease progression profile (red line). The patient required supplemental oxygen but survived the infection.

Hospitalized patients are often subjected to radiographic scanning for their active monitoring. An automatic assessment system capable of comparing successive scans can be highly beneficial to the physicians and radiologists in effectively utilizing their precious time by prioritizing deteriorating patients. In this regard, our proposed method can provide sufficient information to construct a patient progression profile where the first scan is compared with a reference normal CXR and then each successive one is compared with the previous one to determine progression over a period of time using the proposed PEF algorithm. The duration between the two scans, along with the confidence scoring of the proposed system, can be utilized to build the patient profile graph. A few samples of different patients taken from the COVID chest X-ray dataset [23] along with their patient profile graphs are shown in Figure 4, Figure 5, Figure 6 and Figure 7. Each of the patient had three or more successive scans. Their corresponding progression profile graphs shown in the figures below reveal the capability of the proposed method in determining progression (both improvement and deterioration) for active monitoring of hospitalized patients.

The four scans in Figure 4a were taken from a female patient aged 72 years who did not survive the infection. The first scan was taken on the first day of the infection, and subsequent scans were obtained every following day. The patient profile graph indicates consistent deterioration in the CXRs, as shown in Figure 4b. The rapid decline in health of such older patients is quite common with COVID-19 infections, which the proposed method was able to demonstrate in this sample.

The scans in Figure 5a were also obtained from a female patient aged 70 years whose CXRs showed deterioration in the first three scans taken on the days 3, 7, and 9. However, the scan obtained on day 10 exhibited slight improvement, which is depicted in Figure 5b. Similarly, the scans in Figure 6a belong to a male patient aged 74 years. The subsequent scans taken on days 7, 11, and 28 showed consistent improvement over the course of a month. The improvement of the patient was accurately depicted by the proposed method. Similarly, Figure 7a shows the scans obtained from a male patient aged 35 years. Deterioration could be seen in the first three scans taken on days 4, 7, and 9. However, slight improvement was noticed in the scan on day 10. The progression profile accurately depicted the trend, as shown in Figure 7b.

The performance of the proposed method in estimation of disease progression was assessed quantitatively using the test dataset. Scans from 295 patients were included in the evaluation where improvement was observed in 201 patients (68%), deterioration in 77 (26%), and both deterioration and improvement in 17 patients (6%). Table 4 presents the performance metrics of precision, recall, and F-measure. The results indicated that the proposed method was capable of accurately determining progression profile in all the cases. For instance, deterioration and improvement in patients from the test set were detected with 76% and 90% F-scores, respectively, whereas the patients whose scans exhibited both states were detected with 70% F-score.

Experimental results showed that the proposed method could effectively model and recognize patterns in successive radiographic scans for determining progression in COVID-19 patients. Both older and younger patients were tested with the proposed method to observe progression using successive chest X-ray scans. The proposed method shows promise, and further research can improve the overall framework if more significant variables are added to it.

## 4. Discussion

Previous studies have proposed a wide variety of methods to predict progression in COVID-19 patients [4,16,18,19]. However, little attention has been paid to analyze time series observations and modeling of the sequential characteristics of COVID-19 patients. Radiologists regard it as essential to observe the relationship between historical and current observations (vital signs, radiographic scans, physiological characteristics, lab tests, etc.) in order to make informed decisions regarding patient health [16]. It has been found that more accurate predictions regarding disease progression can be made by considering both historical and current readings. In this regard, Sriram et al. [21] studied methods based on both single and multiple scans and found that the sequence-based models outperformed those based on single scans. Their multi-image prediction method yielded superior performance in predicting probabilities of ICU admission, intubation, and mortality. Their approach utilized feature pooling and transformer to perform sequential analysis of multiple CXRs. The authors predicted adverse events with considerable accuracy, but individual patient profiling was not performed. In a similar study, Sun et al. [16] showed that time series analysis of clinical biomarkers can be effectively modeled in order to perform survival prediction in COVID-19 patients. Although they were able to predict disease outcome, they did not utilize radiographic scans. In another study, Jiao et al. [30] proposed a method based on deep learning to predict patient admission, ICU admission, mechanical ventilation, death, and discharge using CXR and clinical data including lab tests and vital signs. However, they used a single image, and the method’s dependency on availability of clinical data means its use is limited in situations where past observations are unavailable.

Several challenges exist when considering sequences of observations (radiographs or other clinical records) for determining progression. Irregular interval between observations and missing values are a few of the problems researchers have to deal with. Deep learning methods have exhibited outstanding performance in prediction tasks. They have also been found to achieve excellent performance in time series prediction. Disease progression detection is intuitively regarded as a sequence observation problem. Therefore, deep sequence learning models are the best candidates for solving this problem. In this work, a method based on sequence learning was proposed and evaluated on COVID-19 datasets to determine disease progression using successive radiographic scans. Deep convolutional features from a pair of scans were utilized by the sequence learning model consisting of GRUs to determine improvement or deterioration. The proposed method was able to predict the change with considerable accuracy. This model forms the basis for a disease progression estimation function that attempts to quantify change and construct disease progression profiles for individual patients. The cases with both deterioration and improvements were more challenging, yet the proposed method was able to detect changes and accurately determine progression.

In most studies, deep learning models are considered as black boxes with very little focus on their interpretability, which restricts their adoption in clinical settings. Medical experts desire interpretable models and are keen to understand how the prediction can be interpreted. In this regard, further work can be carried out with the proposed method to make it interpretable up to some extent. One possibility is to incorporate gradient class activation maps (GradCAM [31]) to visualize biomarkers in the sequence of radiographic scans. It can be helpful for the radiologist if the model can highlight regions where improvement or deterioration has taken place. This will significantly reduce the time needed to prepare the diagnosis report based on the sequence of scans. Similarly, inclusion of other parameters such as comorbidities and other relevant clinical variables into the overall framework will enhance prediction performance of the method. Segmentation-based preprocessing can also be incorporated to further enhance the proposed model. Finally, the mathematical model of the framework needs to be improved so that the progression estimation is carried out on a uniform scale, which would allow better overall management of patients, particularly in emergency situations.

## 5. Conclusions

In this work, we showed that a deep sequence learning model can be used to determine disease progression in COVID-19 CXRs. It was also observed that features extracted from the ChexNet model were superior in their representation ability for COVID-19 CXRs. The proposed method of deriving patient progression profile using positive or negative change in conjunction with the patient age can also be used with other modalities of medical imaging, such as CT scans, MRI, and skin lesion images, to monitor disease progression and even predict prognosis. We believe that our method has promise and that further research is needed to improve the radiographic scan comparison and patient profiling method, which can be helpful in developing an automated triage system for use in unforeseen emergency situations.

One of the shortcomings in the proposed method is that the progression profile is not generated on a uniform scale. Therefore, in its current state, patient progression profiles cannot be directly compared to assess which patient is deteriorating faster compared to the rest. Similarly, we have not considered other variables that add risk to deteriorating patients in COVID-19, such as comorbidities.

In the future, we intend to use multipurpose deep learning methods to automatically quantify improvement or deterioration in successive scans and also predict prognosis. We also plan to use a diverse dataset to fine-tune models for detecting COVID-19-specific abnormalities along with their severity in a single end-to-end sequence learning architecture. Furthermore, integration of other risk factors with the proposed method can reveal interesting insights into disease progression and prognosis prediction of COVID-19 patients.

## Figures and Tables

**Table 1:** Performance evaluation of various feature extractors for image-based prediction.

Model	Feature Dimension	Precision	Recall	F-Measure	AUC
ResNet-50 FT	2048	0.891	0.855	0.873	0.85
InceptionV3 FT	2048	0.883	0.880	0.881	0.88
InceptionResNetV2 FT	1536	0.892	0.892	0.892	0.89
EfficientNet-B0 FT	1280	0.886	0.852	0.869	0.84
EfficientNet-B2 FT	1408	0.871	0.866	0.867	0.87
ChexNet (DenseNet121) PT	1024	0.921	0.918	0.920	0.92

**Table 2:** Performance evaluation of ChexNet features with varying feature extraction approaches.

Granularity	Precision	Recall	F-Measure	AUC
Image-based	0.921	0.918	0.920	0.92
Lung-based	0.959	0.964	0.962	0.96
Zone-based	0.982	0.979	0.981	0.98

**Table 3:** Sequence learning architecture performance for zone-based prediction.

Architecture	Precision	Recall	F-Measure	AUC
1024 GRU + 512 FC + 2 FC	0.982	0.979	0.981	0.98
512 GRU + 512 FC + 2 FC	0.926	0.896	0.911	0.91
1024 GRU + 512 GRU + 128 FC + 2 FC	0.878	0.890	0.883	0.88
1024 GRU + 256 FC + 2 FC	0.944	0.938	0.941	0.94

**Table 4:** Disease progression detection performance using PEF.

Scenario	Bal. Accuracy	Precision	Recall	F-Measure
Deterioration	0.84	0.71	0.82	0.76
Improvement	0.84	0.93	0.87	0.90
Deterioration + Improvement	0.80	0.67	0.73	0.70
Overall	0.82	0.77	0.81	0.78

## Data Availability

Data and relevant material will be made available at: https://github.com/jamilahmadicp/COVID-19-Progression, accessed date 31 October 2021.

## Supplementary Materials

The following are available online at https://www.mdpi.com/article/10.3390/ijerph19010480/s1, Figure S1: Frequency distribution of selected patients’ number of scans in the dataset, Figure S2: Sample scans showing signs of deterioration as infection spreads in a patient from (a) to (c), the area of infected region outgrows the normal lung area in (c), Figure S3: Zone-based segmentation in CXR, Figure S4: Risk factors for age groups, Table S1: Proposed model architecture for sequence learning in CXRs.

## References

[1] Ludwig S., Zarbock A. (2020). Coronaviruses and SARS-CoV-2: A brief overview. Anesth. Analg..

[2] Brown M.J., Goodwin J., Liddell K., Martin S., Palmer S., Firth P., Eyal N., Hyder A., Persad G., Phillips J. (2020). Allocating medical resources in the time of Covid-19. N. Engl. J. Med..

[3] Khan S.A., Manohar M., Khan M., Asad S., Adil S.O. (2021). Radiological profile of patients undergoing Chest X-ray and computed tomography scans during COVID-19 outbreak. Pak. J. Med. Sci..

[4] Zebin T., Rezvy S. (2021). COVID-19 detection and disease progression visualization: Deep learning on chest X-rays for classification and coarse localization. Appl. Intell..

[5] Singh R.K., Pandey R., Babu R.N. (2021). COVIDScreen: Explainable deep learning framework for differential diagnosis of COVID-19 using chest X-rays. Neural Comput. Appl..

[6] Pham H.H., Le T.T., Tran D.Q., Ngo D.T., Nguyen H.Q. (2021). Interpreting chest X-rays via CNNs that exploit hierarchical disease dependencies and uncertainty labels. Neurocomputing.

[7] Zhang J., Xie Y., Pang G., Liao Z., Verjans J., Li W., Sun Z., He J., Li Y., Shen C. (2020). Viral Pneumonia Screening on Chest X-rays Using Confidence-Aware Anomaly Detection. IEEE Trans. Med. Imaging.

[8] Rajpurkar P., Irvin J., Zhu K., Yang B., Mehta H., Duan T., Ding D., Bagul A., Langlotz C., Shpanskaya K. (2017). Chexnet: Radiologist-level pneumonia detection on chest x-rays with deep learning. arXiv.

[9] Esteva A., Kuprel B., Novoa R.A., Ko J., Swetter S.M., Blau H.M., Thrun S. (2017). Dermatologist-level classification of skin cancer with deep neural networks. Nature.

[10] Li Y., Zhang Z., Dai C., Dong Q., Badrigilan S. (2020). Accuracy of deep learning for automated detection of pneumonia using chest X-ray images: A systematic review and meta-analysis. Comput. Biol. Med..

[11] Zheng Q., Lu Y., Lure F., Jaeger S., Lu P. (2020). Clinical and radiological features of novel coronavirus pneumonia. J. X-ray Sci. Technol..

[12] Signoroni A., Savardi M., Benini S., Adami N., Leonardi R., Gibellini P., Vaccher F., Ravanelli M., Borghesi A., Maroldi R. (2020). End-to-end learning for semiquantitative rating of covid-19 severity on chest X-rays. arXiv.

[13] Cohen J.P., Dao L., Roth K., Morrison P., Bengio Y., Abbasi A.F., Shen B., Mahsa H.K., Ghassemi M., Li H. (2020). Predicting covid-19 pneumonia severity on chest x-ray with deep learning. Cureus.

[14] Iandola F., Moskewicz M., Karayev S., Girshick R., Darrell T., Keutzer K. (2014). Densenet: Implementing efficient convnet descriptor pyramids. arXiv.

[15] Fridadar M., Amer R., Gozes O., Nassar J., Greenspan H. (2021). COVID-19 in CXR: From detection and severity scoring to patient disease monitoring. IEEE J. Biomed. Health Inform..

[16] Sun C., Hong S., Song M., Li H., Wang Z. (2021). Predicting COVID-19 disease progression and patient outcomes based on temporal deep learning. BMC Med. Inform. Decis. Mak..

[17] Shamout F.E., Shen Y., Wu N., Kaku A., Park J., Makino T., Jastrzębski S., Witowski J., Wang D., Zhang B. (2021). An artificial intelligence system for predicting the deterioration of COVID-19 patients in the emergency department. NPJ Digit. Med..

[18] Pu J., Leader J.K., Bandos A., Ke S., Wang J., Shi J., Du P., Guo Y., Wenzel S.E., Fuhrman C.R. (2021). Automated quantification of COVID-19 severity and progression using chest CT images. Eur. Radiol..

[19] Feng Z., Yu Q., Yao S., Luo L., Zhou W., Mao X., Li J., Duan J., Yan Z., Yang M. (2020). Early prediction of disease progression in COVID-19 pneumonia patients with chest CT and clinical characteristics. Nat. Commun..

[20] Zhang K., Liu X., Shen J., Li Z., Sang Y., Wu X., Zha Y., Liang W., Wang C., Wang K. (2020). Clinically applicable AI system for accurate diagnosis, quantitative measurements, and prognosis of COVID-19 pneumonia using computed tomography. Cell.

[21] Sriram A., Muckley M., Sinha K., Shamout F., Pineau J., Geras K.J., Azour L., Aphinyanaphongs Y., Yakubova N., Moore W. (2021). COVID-19 Deterioration Prediction via Self-Supervised Representation Learning and Multi-Image Prediction. arXiv.

[22] Vayá M.d.l.I., Saborit J.M., Montell J.A., Pertusa A., Bustos A., Cazorla M., Galant J., Barber X., Orozco-Beltrán D., García-García F. (2020). Bimcv covid-19+: A large annotated dataset of rx and ct images from covid-19 patients. arXiv.

[23] COVID Chest XRay Dataset. https://github.com/ieee8023/covid-chestxray-dataset.

[24] CDC COVID Data Tracker. https://covid.cdc.gov/covid-data-tracker/#demographics.

[25] He K., Zhang X., Ren S., Sun J. Deep residual learning for image recognition. Proceedings of the IEEE Conference on Computer Vision and Pattern Recognition.

[26] Szegedy C., Vanhoucke V., Ioffe S., Shlens J., Wojna Z. Rethinking the inception architecture for computer vision. Proceedings of the IEEE Conference on Computer Vision and Pattern Recognition.

[27] Szegedy C., Ioffe S., Vanhoucke V., Alemi A.A. Inception-v4, inception-resnet and the impact of residual connections on learning. Proceedings of the Thirty-First AAAI Conference on Artificial Intelligence.

[28] Tan M., Le Q. Efficientnet: Rethinking model scaling for convolutional neural networks. Proceedings of the International Conference on Machine Learning.

[29] Extensive COVID-19 X-ray and CT Chest Images Dataset. https://data.mendeley.com/datasets/8h65ywd2jr/3.

[30] Jiao Z., Choi J.W., Halsey K., Tran T.M.L., Hsieh B., Wang D., Eweje F., Wang R., Chang K., Wu J. (2021). Prognostication of patients with COVID-19 using artificial intelligence based on chest x-rays and clinical data: A retrospective study. Lancet Digit. Health.

[31] Selvaraju R.R., Cogswell M., Das A., Vedantam R., Parikh D., Batra D. Grad-cam: Visual explanations from deep networks via gradient-based localization. Proceedings of the IEEE International Conference on Computer Vision.

