# Supplementary material for: Disease Progression Detection via Deep Sequence Learning of Successive Radiographic Scans
Source: Int J Environ Res Public Health. 2022 Jan 2;19(1):480. doi: 10.3390/ijerph19010480 (PMC8744904; doi:10.3390/ijerph19010480)
Supplement: Supplementary file 1 [file ijerph-19-00480-s001.zip › ijerph-1466391-supplementary.pdf]

# Supplementary Material

## 1. Related Work

### *1.1. COVID-19 Detection*

A major portion of the previous works on imaging data of COVID-19 patients focused on detection and diagnosis of the disease from CXRs, and CT scans. Innumerous deep learning-based methods have been developed to improve efficiency and reduce burden on radiologists to interpret CXR images. Wang et al. [1] proposed a weakly supervised method to classify and localize 14 common thoracic diseases exhibiting the capability of CNNs to effectively process CXRs. Investigating further the ability of deep CNNs, Rajpurkar et al. [2] constructed a deep 121-layer dense CNN in order to detect diseases better than expert human radiologists. In another work, Wang et al. [3] utilized attention mechanism to assist the model achieve improved diagnosis performance by focusing on lesion areas in images. Many researchers [4-7] have investigated deep CNNs to detect pneumonia and also differentiate between bacterial and viral pneumonia. Maghdid et al. [8] and Bukhari et al. [9] used AlexNet and ResNet-50 CNN architectures, respectively to classify CXRs as normal or COVID-19 pneumonia with high accuracies. In a similar work, Wang et al. [10] built a custom CNN to detect COVID-19 infection in CXR images. Rajaraman et al. [11] used an iteratively pruned model ensemble for detecting COVID-19 infection in scans. The authors investigated the performance of various deep CNNs on a wide range of datasets and concluded that InceptionV3, VGG19, and VGG16 models performed optimally on the datasets. Hence, their predictions were assigned weights of 0.5, 0.3, and 0.2, respectively. The ensemble of these models achieved the best overall performance. Sedik et al. [12] demonstrated that utilizing data augmentation improves detection performance by 11% whenever data is insufficient. They used a deep convolutional generative adversarial network (DCGAN) to generate augmented images for training deep learning models for COVID-19 detection in CXR. Ismael et al. [13] reviewed many deep learning models for detection in CXR images and showed that fine-tuning recent deep CNNs on COVID-19 datasets achieves excellent detection performance. Their evaluation showed that using a fine-tuned ResNet-50 model as a feature extractor with SVM classifier yielded optimal performance. In a similar study, Luz et al. [14] fine-tuned EfficientNet model to detect COVID-19 in CXRs. These models are constructed automatically by combining optimal units to achieve the best performance at low cost. Their model can detect COVID-19 pneumonia and can also differentiate it from non-COVID pneumonia. They also evaluated their model on a different dataset to show that the model express generalization. Tackling the problem of insufficient data, Gupta et al. [15] also fine-tuned a number of pre-trained deep CNNs and achieved high detection accuracy. Zhang et al. developed an anomaly detection system where they attempted to spot novel abnormalities as anomalies from CXRs. Their system learned existing pulmonary disorders and their confidence scoring mechanism reports anomaly when an unseen pattern is perceived. Recent works utilizing deep learning with sufficiently large datasets, data augmentation to expand smaller datasets, and fine-tuning pre-trained CNNs, exhibited the capability of these models to accurately detect abnormalities in CXR images. These algorithms are capable of performing advanced pattern recognition tasks with a high degree of accuracy if appropriately prepared data is fed to them in sufficient amounts.

### *1.2. Severity Estimation*

In addition to disease detection in CXRs, many researchers have estimated the severity of infection in CXR scans using deep CNNs. In a recent study Signoroni et al. [16] developed a multi-purpose network to detect COVID-19 pneumonia, segment and align lung regions, output severity scores by dividing the lungs into six regions. A regression head was trained on a large dataset with severity scores provided by expert radiologists for the purpose of estimating disease severity. The model achieved a mean absolute error (MAE) of 1.8. In a similar study, Cohen et al. [17] pre-trained a DenseNet [18] model on 18 common radiological findings from several publicly available datasets. A linear regression model was then trained on severity scores for pneumonia extent and opacity scores provided by three expert radiologists. Amer et al. [19] trained a deep learning model to simultaneously train a detection and localization model for pneumonia in CXRs. The localization maps were then used to estimate pneumonia ratio indicating severity

of the infection. Blain et al. [20] used a similar approach where they trained a U-Net model to segment lungs and a DenseNet121 model to detect lung abnormalities like interstitial and alveolar opacity. Information from both models was then used to estimate disease severity in CXRs. These studies showed that the severity scoring allowed hospitals to maintain a disease severity profile for each patient to monitor their progress.

## 2. Materials and Methods

### 2.1. Datasets

The frequency distribution of the BIMCV dataset is provided in Figure S1.

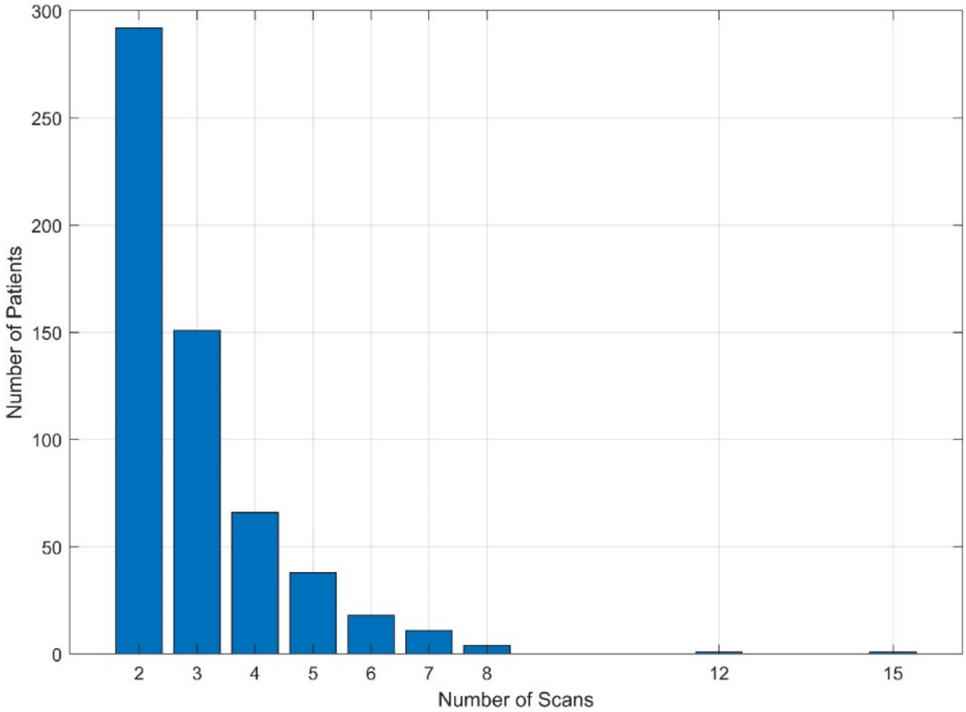


**Figure S1.** Frequency distribution of selected patients' number of scans in the dataset.

### 2.2. Visual Changes in CXR in Response to COVID-19

A normal CXR does not have any visual symptoms corresponding to any pathologies. However, when a patient is infected with COVID-19, the CXR begin to show sign of infection in the form of infiltrates and opacities along with other abnormalities. As the disease progresses over time, these visual symptoms either fade away (improvement) or get intensified (deterioration). As the infection spreads in COVID-19 patients, the shadowing (i.e., white portion) grows inside the lung region. If the area of the abnormal lung (white region) is relatively smaller, the infection is regarded as "mild". When the abnormal lung area grows to cover half of the lung region, it is considered as "moderate infection", whereas a further spread is then classified as "severe". These visual cues are thoroughly read by the radiologist to assess the severity of infection. It can be seen from the radiographs in Figure S2 that the deterioration in successive scans is quite clear in terms of visual cues as the infection spread in the lungs.

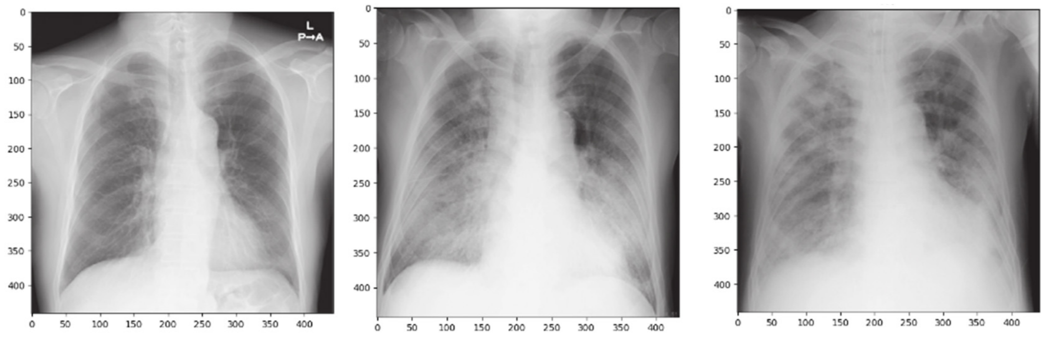

(a) (b) (c)

**Figure S2.** Sample scans showing signs of deterioration as infection spreads in a patient from (a) to (c), the area of infected region outgrows the normal lung area in (c) [24].

## *2.3. Image Annotation Schemes*

Three different schemes were devised for sequence modeling of successive CXRs, i.e., 1) image-based, 2) lung-based, and 3) zone-based. In image-based scheme, each scan was consumed as a whole by the features extraction module. The sequence in this case consisted of two images. In the lung-based method, left and right lungs of both images were separated and a sequence of 4 patches (lungs) was constructed. Lastly, each lung was further divided into three zones (upper, middle, and lower) as shown in Figure S3, where the image sequence consisted of 12 patches in total. In either strategy, the label was either deterioration, improvement, or no-change.

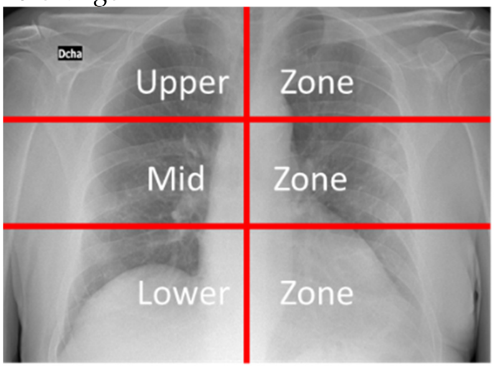


**Figure S3.** Zone-based segmentation in CXR.

## *2.4. Gated Recurrent Units*

Gated recurrent units (GRUs) are an improved form of vanilla recurrent neural networks (RNN). They are similar in function to the long short-term memory (LSTM) in their use of gates to control the flow of information. However, GRUs are simpler in construction to LSTM cells, and therefore contain fewer parameters, thereby making them relatively faster to train compared to LSTM. A typical GRU consist of several components including update gate, reset gate, current memory content, and final memory at current time step. The update gate is responsible for passing past information to the future. It is indicated as

$$z_t = \sigma(W^{(z)} F_{seq}^{t} + U^{(z)} h_{t-1}) \quad \text{(S1)}$$

Where W and U are the weights for current input $F_{seq}^{t}$ and previous input $h_{t-1}$, sigma is the activation function and $z_t$ is the output. The reset gate, having similar calculation method, possess different weights which allow it to decide how much of the previous information needs to be forgotten.

$$r_t = \sigma(W^{(r)} F_{seq}^{t} + U^{(r)} h_{t-1}) \quad \text{(S2)}$$

The current memory content, computed as (S3) where the $r_t$ determines which parts of the previous information $h_{t-1}$ will be forgotten and which will be kept which is achieved by an element-wise multiplication operation.

$$h_t^{'} = \tanh(W.F_{seq}^{t} + r_t \odot U h_{t-1}) \quad \text{(S3)}$$

The final memory at current time step is computed as (S4) determines how much to collect from current memory content $h_t$ and from the previous steps $h_{t-1}$.

$$h_t = z_t \odot h_{t-1} + (1 - z_t) \odot h_t^{'} \quad \text{(S4)}$$

*2.5. Progression and severity detection*

The risk factor scores for this study depicted in Figure S4 were calculated from CDC data for the US [24].

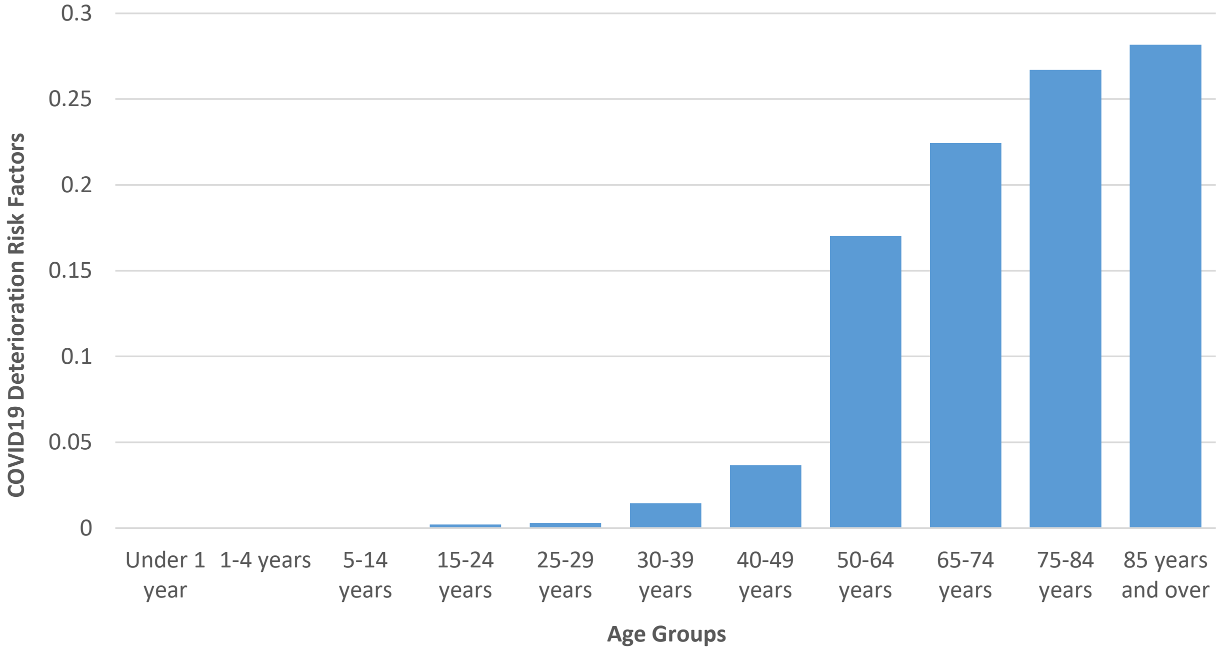


**Figure S4.** Risk factors for age groups.

*2.6. Model Architecture & Training*

The layers forming the features extraction network are kept frozen during training. The sequence learning network is trained for 30 epochs with a batch size of 32. Adam optimizer with an initial learning rate of 0.0001, and binary cross entropy as loss function was used to optimize the parameters.

**Table S1.** Proposed model architecture for sequence learning in CXRs.

| Layer | Output | Parameters |
|---|---|---|
| Input Layer | N × (224, 224, 3) | |
| Features Extraction Layer (ChexNet) | N × 1024 | 7,037,504 |
| GRU | 1024 | 6,297,600 |
| Dense | 512 | 524,800 |
| Dropout | 512 | |
| Classification Layer | 2 | 1026 |
| | Total | 13,860,930 |

**References**


1. Wang, X.; Peng, Y.; Lu, L.; Lu, Z.; Bagheri, M.; Summers, R.M. Chestx-ray8: Hospital-scale chest x-ray database and benchmarks on weakly-supervised classification and localization of common thorax diseases. In Proceedings of Proceedings of the IEEE conference on computer vision and pattern recognition; pp. 2097-2106.
2. Rajpurkar, P.; Irvin, J.; Zhu, K.; Yang, B.; Mehta, H.; Duan, T.; Ding, D.; Bagul, A.; Langlotz, C.; Shpanskaya, K. Chexnet: Radiologist-level pneumonia detection on chest x-rays with deep learning. *arXiv preprint arXiv:1711.05225* **2017**.
3. Wang, H.; Jia, H.; Lu, L.; Xia, Y. Thorax-net: an attention regularized deep neural network for classification of thoracic diseases on chest radiography. *IEEE journal of biomedical and health informatics* **2019**, *24*, 475-485.
4. Kermany, D.S.; Goldbaum, M.; Cai, W.; Valentim, C.C.; Liang, H.; Baxter, S.L.; McKeown, A.; Yang, G.; Wu, X.; Yan, F. Identifying medical diagnoses and treatable diseases by image-based deep learning. *Cell* **2018**, *172*, 1122-1131. e1129.
5. Rajaraman, S.; Candemir, S.; Kim, I.; Thoma, G.; Antani, S. Visualization and interpretation of convolutional neural network predictions in detecting pneumonia in pediatric chest radiographs. *Applied Sciences* **2018**, *8*, 1715.
6. Ibrahim, A.U.; Ozsoz, M.; Serte, S.; Al-Turjman, F.; Yakoi, P.S. Pneumonia classification using deep learning from chest X-ray images during COVID-19. *Cognitive Computation* **2021**, 1-13.
7. Acharya, A.K.; Satapathy, R. A deep learning based approach towards the automatic diagnosis of pneumonia from chest radio-graphs. *Biomedical and Pharmacology Journal* **2020**, *13*, 449-455.
8. Maghdid, H.S.; Asaad, A.T.; Ghafoor, K.Z.; Sadiq, A.S.; Mirjalili, S.; Khan, M.K. Diagnosing COVID-19 pneumonia from X-ray and CT images using deep learning and transfer learning algorithms. In Proceedings of Multimodal Image Exploitation and Learning 2021; p. 117340E.
9. Bukhari, S.U.K.; Bukhari, S.S.K.; Syed, A.; Shah, S.S.H. The diagnostic evaluation of Convolutional Neural Network (CNN) for the assessment of chest X-ray of patients infected with COVID-19. *MedRxiv* **2020**.
10. Wang, L.; Lin, Z.Q.; Wong, A. Covid-net: A tailored deep convolutional neural network design for detection of covid-19 cases from chest x-ray images. *Scientific Reports* **2020**, *10*, 1-12.
11. Rajaraman, S.; Siegelman, J.; Alderson, P.O.; Folio, L.S.; Folio, L.R.; Antani, S.K. Iteratively pruned deep learning ensembles for COVID-19 detection in chest X-rays. *IEEE Access* **2020**, *8*, 115041-115050.
12. Sedik, A.; Iliyasu, A.M.; El-Rahiem, A.; Abdel Samea, M.E.; Abdel-Raheem, A.; Hammad, M.; Peng, J.; El-Samie, A.; Fathi, E.; El-Latif, A. Deploying machine and deep learning models for efficient data-augmented detection of COVID-19 infections. *Viruses* **2020**, *12*, 769.
13. Ismael, A.M.; Şengür, A. Deep learning approaches for COVID-19 detection based on chest X-ray images. *Expert Systems with Applications* **2021**, *164*, 114054.
14. Luz, E.; Silva, P.; Silva, R.; Silva, L.; Guimarães, J.; Miozzo, G.; Moreira, G.; Menotti, D. Towards an effective and efficient deep learning model for COVID-19 patterns detection in X-ray images. *Research on Biomedical Engineering* **2021**, 1-14.
15. Gupta, A.; Gupta, S.; Katarya, R. InstaCovNet-19: A deep learning classification model for the detection of COVID-19 patients using Chest X-ray. *Applied Soft Computing* **2021**, *99*, 106859.

16. Signoroni, A.; Savardi, M.; Benini, S.; Adami, N.; Leonardi, R.; Gibellini, P.; Vaccher, F.; Ravanelli, M.; Borghesi, A.; Maroldi, R. End-to-end learning for semiquantitative rating of covid-19 severity on chest x-rays. *arXiv preprint arXiv:2006.04603* **2020**, *6*.
17. Cohen, J.P.; Dao, L.; Roth, K.; Morrison, P.; Bengio, Y.; Abbasi, A.F.; Shen, B.; Mahsa, H.K.; Ghassemi, M.; Li, H. Predicting covid-19 pneumonia severity on chest x-ray with deep learning. *Cureus* **2020**, *12*.
18. Iandola, F.; Moskewicz, M.; Karayev, S.; Girshick, R.; Darrell, T.; Keutzer, K. Densenet: Implementing efficient convnet descriptor pyramids. *arXiv preprint arXiv:1404.1869* **2014**.
19. Fridadar, M.; Amer, R.; Gozes, O.; Nassar, J.; Greenspan, H. COVID-19 in CXR: From detection and severity scoring to patient disease monitoring. *IEEE journal of biomedical and health informatics* **2021**.
20. Blain, M.; Kassin, M.T.; Varble, N.; Wang, X.; Xu, Z.; Xu, D.; Carrafiello, G.; Vespro, V.; Stellato, E.; Ierardi, A.M. Determination of disease severity in COVID-19 patients using deep learning in chest X-ray images. *Diagnostic and interventional radiology* **2021**, *27*, 20.
